# Human leukocyte antigen class I supertypes and viral control in HIV-1 infected former plasma donors from China

**DOI:** 10.1186/1742-4690-9-S2-P167

**Published:** 2012-09-13

**Authors:** J Hao, K Hong, J Chen, M Jia, Y Ruan, Y Shao

**Affiliations:** 1 National Center for AIDS/STD Control and Prevention, Beijing, China

## Background

The role of human leukocyte antigen (HLA) class I supertypes in controlling human immunodeficiency virus type 1 (HIV-1) infection in Chinese has not been established. The aim of this study is to examine the frequency of HLA-A and HLA-B alleles and supertypes of 222 HIV-1 infected former plasma donors in central China and to investigate their impact on HIV-1 viral control.

## Methods

HLA-A and HLA-B alleles were genotyped with PCR-SSP and sequence-based typing assay to four-digit resolution. The HLA alleles were classified functionally to 4 HLA-A supertypes and 6 HLA-B supertypes according to their shared peptide binding properties. Plasma viral load was determined using the Roche Amplicor ultrasensitive assay which has a lower detection limit of 50 copies HIV-1 RNA per ml.

## Results

HLA-A03 supertypes(A03s) and HLA-B62 supertypes(B62s) were associated with lower viral load (P=0.0206, P=0.0483), whereas HLA- A24 supertypes(A24s) appeared to have an association with higher viral load (P=0.0483). There was a highly significant correlation between the genotypic supertypes(GS) and viral load (Kendall's tau b = 0.180, P=0.000). The median viral load was lower among A*3001(P=0.0139), A*1101(P=0.0096), B*5101(P=0.0025), B*3501(P=0.0091) or B*4601(P=0.001) carriers and higher in A*2301(P=0.0106) carriers.

## Conclusion

HLA-A03s and -B62s may be associated with favorable HIV-1 viral control, A24s associated with unfavorable viral control; HLA-B*4601 within B62s and HLA-A*2301 within A24s might contribute to the outcomes of HIV-1 viral control.

